# Structural Transitions of Papain-like Cysteine Proteases: Implications for Sensor Development

**DOI:** 10.3390/biomimetics8030281

**Published:** 2023-07-01

**Authors:** Srdjan Marković, Natalija S. Andrejević, Jelica Milošević, Natalija Đ. Polović

**Affiliations:** Faculty of Chemistry, University of Belgrade, Studentski trg 12-16, 11000 Belgrade, Serbia

**Keywords:** papain, cystein protease, S-Methyl methanethiosulfonate, sensor, covalent modification, conformational analysis, protein stability

## Abstract

The significant role of papain-like cysteine proteases, including papain, cathepsin L and SARS-CoV-2 PLpro, in biomedicine and biotechnology makes them interesting model systems for sensor development. These enzymes have a free thiol group that is suitable for many sensor designs including strong binding to gold nanoparticles or low-molecular-weight inhibitors. Focusing on the importance of the preservation of native protein structure for inhibitor-binding and molecular-imprinting, which has been applied in some efficient examples of sensor development, the aim of this work was to examine the effects of the free-thiol-group’s reversible blocking on papain denaturation that is the basis of its activity loss and aggregation. To utilize biophysical methods common in protein structural transitions characterization, such as fluorimetry and high-resolution infrared spectroscopy, low-molecular-weight electrophilic thiol blocking reagent S-Methyl methanethiosulfonate (MMTS) was used in solution. MMTS binding led to a two-fold increase in 8-Anilinonaphthalene-1-sulfonic acid fluorescence, indicating increased hydrophobic residue exposure. A more in-depth analysis showed significant transitions on the secondary structure level upon MMTS binding, mostly characterized by the lowered content of α-helices and unordered structures (either for approximately one third), and the increase in aggregation-specific β-sheets (from 25 to 52%) in a dose-dependant manner. The recovery of this inhibited protein showed that reversibility of inhibition is accompanied by reversibility of protein denaturation. Nevertheless, a 100-fold molar excess of the inhibitor led to the incomplete recovery of proteolytic activity, which can be explained by irreversible denaturation. The structural stability of the C-terminal β-sheet rich domain of the papain-like cysteine protease family opens up an interesting possibility to use its foldamers as a strategy for sensor development and other multiple potential applications that rely on the great commercial value of papain-like cysteine proteases.

## 1. Introduction

Thiol proteases are ubiquitous in nature, being found in all living organisms. Their physiological role involves the proteolysis of various substrates in a vast number of physiological processes, such as digestion [1], blood coagulation [2], defense and protein turnover [3], cell signaling, and differentiation [4]. Their activity has been associated with many human diseases, including Alzheimer’s disease [5], arthritis [6], cardiovascular diseases [7], and inflammation [8]. Furthermore, many pathogens depend on thiol proteases for a successful lifecycle, including parasites that cause tropical diseases (malaria [9], Schistosomiasis [10], Leishmaniasis and Trypanosomiasis [11] and viruses that cause severe syndromes such as severe acute respiratory syndrome (SARS) [12] and Middle East respiratory syndrome (MERS) [13]. 

Noting the importance of thiol proteases in various fields of biomedicine, papain-like cysteine proteases, including papain, cathepsin L and SARS-CoV-2 PLpro, have been used as model systems to test and validate sensors for the determination of proteolytic activity [14,15,16,17]. By immobilizing magnetic nanoparticles to the surface of a giant magnetoresistive spin-valve sensor via specific synthetic substrate peptides, Adem and coauthors determined the proteolytic activity of papain in real-time [14]. A cheaper and more prominent strategy has been developed by using either natural or synthetic immobilized inhibitors to capture papain-like proteases. This strategy is based on the specific interaction between a protease and its inhibitor and was used for the detection of papain [15], cathepsin L [16], and SARS-CoV-2 PLpro [17] via surface plasmon resonance sensors. Recently, molecularly imprinted polymers were used for the selective detection of papain at trace levels. This kind of sensor could capture the target protein selectively, causing the enhancement of resonance light-scattering intensity with the increase in papain concentration [18].

As it is easily accessible in high amounts, plant, and microbial thiol proteases are among the most commonly used enzymes in biotechnology. The most commonly used thiol proteases are papain-like cysteine proteases due to their wide substrate specificities and high proteolytic activity. Applications include meat tenderization, the removal of dental caries, the production of significant antibody fragments, and utilization as an ingredient in cosmetic products and detergent formulations [19]. Noting the biotechnological and the biomedical applications of papain-like cysteine proteases, traces of those could be found in the environment, pharmaceutical, chemical, and food products, increasing the need to examine different strategies in sensor developments to detect papain-like cysteine proteases. In addition, papain has been further utilized for sensors sensing heavy metal ions [20,21], and bioactive food ingredients [22], and even proposed as a candidate for the determination of tetrahydrocannabinol derivates in urine [23]. In those cases, gold nanoparticles were functionalized with papain due to the strong binding between the cysteine thiol group and gold [24]. This kind of coupling is usually accompanied by some loss in proteolytic activity [25], aggregation and precipitation [26]. 

As the free thiol group in papain-like cysteine proteases is a common target for coupling, and the preservation of the native fold is essential for inhibitor binding and molecular imprinting, it is of great importance for sensor development to investigate if thiol reactions affect protein conformation. The aim of this work was thus to examine the effects of free thiol group reversible blocking on papain structural transitions, which is accompanied by activity loss and aggregation. Biophysical methods that are commonly used in the characterization of protein structural transitions, such as fluorimetry and high-resolution infrared spectroscopy, were applied to monitor changes induced by low-molecular-weight electrophilic thiol blocking reagent S-Methyl methanethiosulfonate (MMTS) [27] binding in solution. The reversibility of the active site thiol blocking and consequent enzyme inhibition enabled the investigation of the reversibility of structural changes, providing insights into the stability and robustness of papain-like cysteine proteases that are important to sensor design.

## 2. Materials and Methods

### 2.1. Papain Purification

Papain was isolated according to the previously published protocol [19] with minor modifications. Briefly, 36 g of papaya leaf powder was resuspended in 240 mL of 100 mM acetate buffer pH 5, and the mixture was shaken for 30 min, after which it was centrifuged at 4000× *g* for 3 min. The solution was separated by straining through dry, well-packed cotton wool, the pH value of the solution was adjusted to 5, and 32 g of (NH_4_)_2_SO_4_ (corresponding to 30% of (NH_4_)_2_SO_4_ saturation) was added to the 144 mL of the resulting solution and further centrifuged at 4000× *g* for 5 min. The supernatant was separated by decantation, another 32 g of (NH_4_)_2_SO_4_ (corresponding to the total of 60% of (NH_4_)_2_SO_4_ saturation) was added, and it was left to cool in the freezer for 10 min. The mixture was then centrifuged for 5 min at 4000× *g* and the supernatant was separated by decantation while the precipitate was dissolved in 36 mL of 1 mM acetate buffer pH 5. A total of 36 mL of purified papain solution in 1 mg/mL concentration was obtained. The solution was dialyzed 2 times for 20 min against 100 mM tris(hydroxymethyl)aminomethane (Tris) buffer pH 8. 

### 2.2. Bradford Microplate Assay for Determining Protein Concentration

To determine the protein concentration, a microplate version [19] of the Bradford assay [28] was used. In brief, a mixture of 5 μL of the sample and 200 μL of the Bradford reagent was prepared in a microplate well, and the absorbance was measured in triplicate after 10 min at 620 nm using LKB Micro plate reader 5060-006. A calibration curve (r^2^ = 0.9901) is presented in the Supplementary Materials with a linearity range of 0.1–1 mg/mL of bovine serum albumin (BSA).

### 2.3. Determination of Proteolytic Activity 

The synthetic substrate Nα-Benzoyl-DL-arginine 4-nitroanilide hydrochloride (BAPNA) was employed to measure proteolytic activity since the activity recovery was shown to be a reliable parameter for sensing the combined effect of multiple stressors (such as structural changes, aggregation and/or autolysis) on proteases, with repeatability for differently treated samples ranging from 3.0 to 14.1% [29]. In summary, 50 μL of the test sample, along with 50 μL of 1 M Tris buffer pH 8, and 100 μL of 0.1% BAPNA solution in 100 mM Tris buffer pH 8 with 5% dimethyl sulfoxide (DMSO), were combined in a 1.5 mL Eppendorf tube. To the blank sample, 50 μL of 10% trichloroacetic acid (TCA) was added to prevent activity. The samples and the blank were incubated at 50 °C for 1 h. The enzyme was inactivated by the addition of 50 μL of 10% TCA to all samples except the blank. After cooling, solutions were centrifuged at 9000× *g* for 5 min, and then 200 μL of the clear solution was transferred to the wells of a microplate. The samples were analyzed for absorbance at a wavelength of 405 nm, and the tests were carried out in triplicate. Errors are presented as relative standard deviations of triplicates. To prevent interday influence on possible autolytic, storage-related or aggregation-induced activity loss [29], the activity of the samples was measured on the same day following purification and MMTS treatment.

### 2.4. MMTS Treatment of Papain and Its Activity Recovery

A total of 100 µL of 1, 10, and 100 mM MMTS solution in water were added to four samples containing 1 mL of purified papain solution resulting in 1:1, 1:10, and 1:100 protein to reagent molar ratios, respectively. Solutions were gently shaken and allowed to incubate at room temperature (RT) for 30 min. The activity was recovered by adding 100 µL of 200 mM dithiothreitol (DTT) solution in water to each sample and incubating them at RT for 30 min. 

### 2.5. Structural Characterization of MMTS-Treated Papain 

#### 2.5.1. 8-Anilinonaphthalene-1-sulfonic Acid (ANS) Fluorescence

A total of 190 µL of 100 mM Tris buffer pH 8, 10 µL of sample, and 20 µL of 10 mM ANS solution in 100 mM Tris buffer pH 8 were applied to the wells of the microplates. Samples were irradiated with light of wavelength 390 nm and the fluorescence emission was recorded at 480 nm. The assay was successfully used in sensing structural transitions of proteins related to hydrophobic core exposure, with repeatability for differently treated samples ranging from 2.5 to 15.4% [29,30]. The tests were carried out in pentaplicate. Errors are presented as relative standard deviations of pentaplicates. To prevent interday influence on possible autolytic, storage-related, and aggregation-induced changes in water exposure of hydrophobic regions [29], fluorescence was measured on the same day following purification and MMTS treatment.

#### 2.5.2. Sodium Dodecylsulfate Polyacrylamide Gel Electrophoresis (SDS-PAGE)

For SDS-PAGE analysis, 12% running gels and 4% stacking gels were prepared. Gels were prepared according to the original procedure [31]. Samples were prepared and run in denaturing and reducing conditions, as described by Markovic and coauthors [19]. To avoid disulfide reduction and acive-site regeneration, samples were prepared in non-reducing conditions (β-mercaptoetanol was not added to the sample buffer) by mixing 4 volumes of samples with 1 volume of 5× concentrated non-reducing sample buffer prior to boiling for 15 min. A total of 12 µg of protein per lane was applied.

#### 2.5.3. Fourier Transform Infrared Spectroscopy (FTIR) and Secondary Structures Calculation

The Nicolet 6700 FTIR spectrometer (Thermo Scientific, Carlsbad, CA, USA) was used to record Infrared (IR) spectra of papain samples in attenuated total reflectance (ATR) mode at 1 cm^−1^ resolution. Samples were prepared by applying 3 µL of papain solution to the diamond of the crystal and drying the applied sample by passing a nitrogen current over it. A total of 64 scans were collected to record each spectrum. To correct the raw spectra, ATR correction, automatic baseline correction, and automatic smoothing were applied using OMNIC 7.3 software.

Amide I regions (frequencies 1700–1600 cm^−1^) of the recorded spectra were decomposed to their peak constituents by calculating Savitzky-Golay second derivative spectra and applying Fourier self-deconvolution using OMNIC software. The Amide I region’s total area under the curves was computed by adding the areas of each individual peak, similar to previously reported methods [32,33]. The assignment of secondary structures for all the observed peaks was carried out according to the previously published guidelines [19,32,33,34,35]. The chain without a signal peptide and propeptide (the UniProt entry [P14080, PAPA2_CARPA]) was used to calculate the secondary structure content within the crystal structure of the mature enzyme.

## 3. Results

### 3.1. Papain Treatment and Recovery

**Figure 1:**
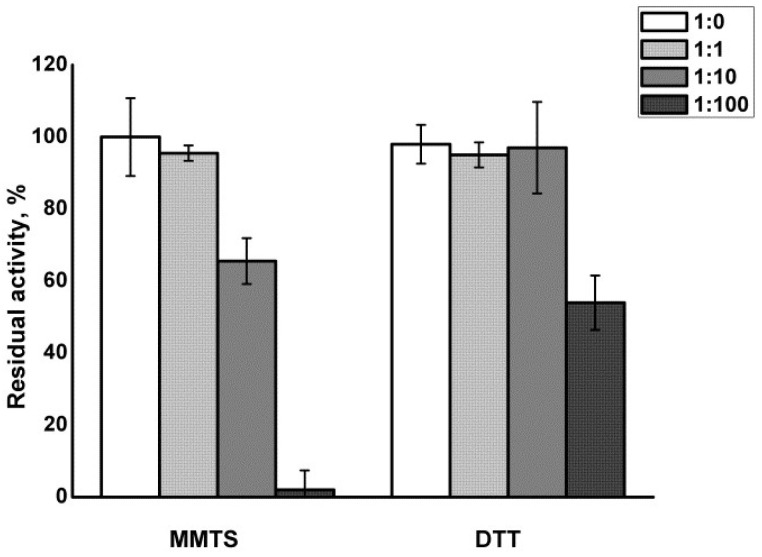
Residual activity of papain after blocking of the free thiol group with MMTS, and its recovery with DTT treatment. Molar ratios papain:MMTS were in the range from 1:0 to 1:100. Values are presented as the averages of triplicates, and the error bars show the relative standard deviations.

Figure 1 shows that MMTS treatment at molar ratios of 1:1 did not significantly influence the residual enzymatic activity of papain. MMTS added in molar excess 1:10 led to only moderate (about 35%) inhibition of the enzyme, with full recovery of the activity after the addition of DTT. Full inhibition of the enzyme was achieved only in 100-fold molar excess of MMTS. However, the recovery of enzymatic activity upon treatment with DTT was only about 55%.

### 3.2. Structural Transitions and Aggregation

#### 3.2.1. Monitoring of Water-Exposed Hydrophobic Regions

ANS is a fluorophore that shows an increase in fluorescence upon binding to water-exposed hydrophobic patches on protein molecules. It can thus sense structural perturbations in the protein, leading to the increased exposure of hydrophobic regions to water [29,30]. 

**Figure 2:**
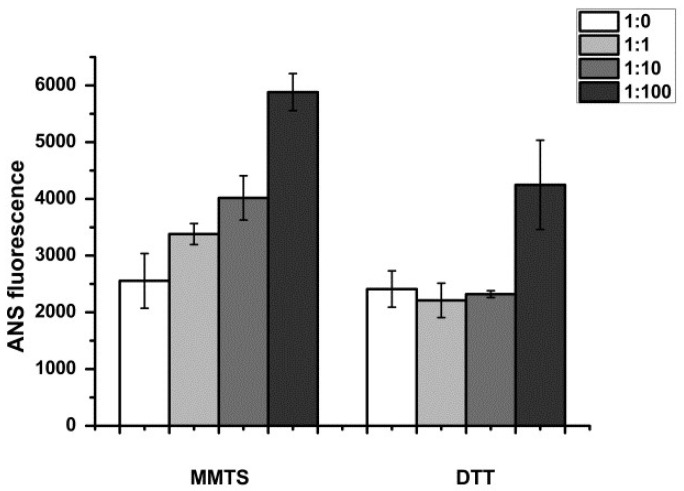
ANS fluorescence of papain after blocking of the free thiol group with MMTS, and its recovery with DTT treatment. Molar ratios papain:MMTS were in the range from 1:0 to 1:100. Values are presented as the averages of pentaplicates, and the error bars show the relative standard deviations.

ANS fluorescence was increased in papain upon MMTS treatment ranging from a 1.4-fold to about 2-fold increase, proportionally to the molar excess of the thiol blocking reagent used, indicating increased hydrophobic residue exposure upon active site blocking (Figure 1). The unfolding of papain was partially irreversible at the 100-fold molar excess of MMTS (Figure 2). 

#### 3.2.2. Papain Aggregation

**Figure 3:**
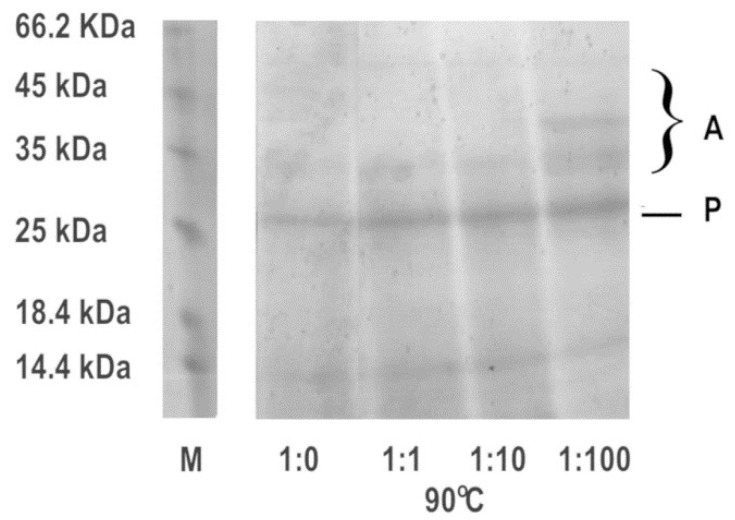
SDS PAGE of papain after blocking of the free thiol group with MMTS, and its recovery with DTT treatment. Samples were prepared by heating at 90 °C for 15 min. Molar ratios papain:MMTS were in the range from 1:0 to 1:100. P—papain; A—aggregates.

Since irreversible unfolding is usually accompanied by the aggregation of misfolded polypeptides, the MMTS-treated enzyme samples were analyzed by SDS PAGE (Figure 3). 

Samples showed several discrete bands in electrophoretic analysis. A band around 25 kDa represents papain, while bands of higher molecular weight could be attributed to aggregates that are resistant to thermal solubilization even in the presence of detergent SDS. 

#### 3.2.3. Transitions of Secondary Structures

**Figure 4:**
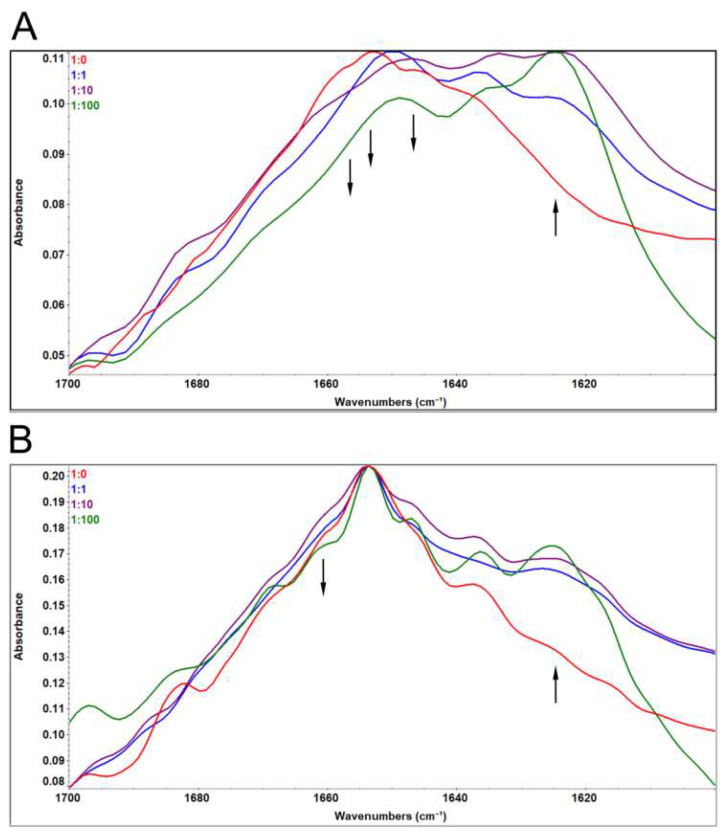
FTIR spectroscopy of papain after blocking of the free thiol group with MMTS: (**A**) without DTT recovery; (**B**) after DTT recovery. Molar ratios papain:MMTS were in the range from 1:0 to 1:100. Arrows are representing trends of changes in spectra.

FTIR was used to investigate the changes in secondary structures. The bands in the Amide I region of the spectrum primarily arise from the C=O-stretching vibrations of amide groups, making it the most sensitive spectral region to changes in the polypeptide backbone conformation [19,32,33]. Figure 4 displays the spectra of MMTS-treated samples, with or without recovery with DTT. The most prominent changes in papain Amide I region induced by treatment with MMTS occurred in a dose-dependent manner, and they are a reduction in the band with three maxima at around 1653 cm^−1^ assigned to α-helix, and around 1660 and 1643 cm^−1^ assigned to unordered structures, while the band around 1623 cm^−1^ corresponding to aggregation-specific β-sheet increased (Figure 4A). 

Treatment with DTT recovered most of the native-like structures, except the unordered band at 1660 cm^−1^, while the β-sheet band specific to aggregation at around 1623 cm^−1^ remained partly increased (Figure 4B). 

The secondary structures’ content was determined by analyzing the Amide I region using deconvolution (Table 1).

β-sheet content increases from 25.5 to 52.0% in MMTS-treated samples in a dose-dependent manner, mostly due to the decrease in unordered structures (from 43.0 to 28.2%) and α-helix content (from 24.9 to 14.6%). At the highest concentration of MMTS used, even after DTT treatment, β-sheet content remained elevated by approximately 5% in comparison to the starting sample, due to the decrease in unordered structures. 

## 4. Discussion

**Figure 5:**
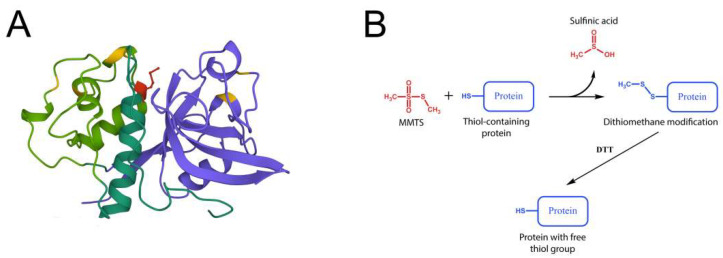
(**A**) 3D Structure of papain (PDB 1YAL), N-terminal domain is shown in green (unstable sequence region 47-107—light green), C-terminal domain in purple, active site Cys25—red, other 6 Cys forming disulfide bridges—yellow; (**B**) schematic representation of MMTS—thiol reaction.

Mature papain consists of 212 amino acid residues organized in two domains—N-terminal α-helix rich domain and C-terminal β-sheet rich domain, with both of them possessing an inner hydrophobic core and surface-exposed polar amino acid residues. The active site of papain is located at the interface between the two domains, and it contains a catalytic triad of amino acids: cysteine, histidine, and asparagine. The cysteine residue (Cys25) is located in the active site cleft and functions as a nucleophile in the catalytic mechanism. The histidine residue (His159) acts as a general acid-base catalyst, while the asparagine residue (Asn175) stabilizes the histidine residue and helps to orient the substrate [36] (Figure 5A).

In this work, MMTS was successfully used to reversibly inhibit papain by forming a disulfide bond with Cys25 from the enzyme’s active site (Figure 5B). The reaction mechanism involves the modification of the thiol group of the cysteine residue in the active site of the enzyme with a methylthio group (-SCH_3_), resulting in the formation of a covalent adduct between MMTS and the enzyme, which can then be activated using reducing agents such as DTT. The reaction mechanism of MMTS with cysteine proteases can be divided into three steps. Firstly, the thiol group of the cysteine residue in the active site of the enzyme attacks the electrophilic sulfur atom in the MMTS molecule. This results in the formation of an intermediate complex, in which the sulfur atom of MMTS is covalently bound to the sulfur atom of the thiol group of the cysteine residue. Then, the intermediate complex undergoes alkylation, in which the methyl group from the methylthio group of MMTS is transferred to the thiol group of the cysteine residue. This results in the formation of a covalent adduct between MMTS and the cysteine residue. The covalent adduct between MMTS and the cysteine residue in the active site of the enzyme blocks the enzyme’s catalytic activity. The inhibition is reversible, and finally, the adduct can be removed by treatment with a reducing agent such as DTT, which reduces the disulfide bond formed between the cysteine residue and the MMTS [37].

The activity was recovered after reduction with DTT, with the exception of the sample that was inhibited with a 100-fold molar excess of MMTS (Figure 1). The results are in line with recently published studies where MMTS was used to inhibit either thiol proteases or other free-thiol-group-dependent enzymes such as dehydrogenases and isomerases [38,39]. MMTS binding led to increased ANS fluorescence, indicating structural perturbations and an increase in the water-exposed hydrophobic surface of the treated protein (Figure 2). ANS fluorescence recovered after treatment with DTT if lower concentrations of MMTS were used, suggesting the fast-refolding and only partial and reversible denaturation of papain. In the previously reported results, there was a significant five-fold increase in the ANS fluorescence when the ovalbumin protein was completely denatured [32], while, in the case of completely blocked papain in our study, the increase in ANS fluorescence was around two-fold. Comparing the ANS fluorescence of the sample in which the inhibition was complete to the ANS fluorescence in the completely thermally denatured ovalbumin, it can be concluded that while papain enzyme’s activity is abolished, it is only being denatured to some extent. The fact that denaturation is not complete indicates that only a part of the protein is destabilized when the active site is being covalentl” mod’fied. Similarly, the destabilization of trypsin following seven freeze–thaw cycles to the molten globe intermediate state led to an even slighter increase in ANS fluorescence (about 1.2-fold) [29]. Only in the case of the 100-fold molar excess of the MMTS did the incomplete recovery of the activity, together with the increased ANS fluorescence (Figure 1 and Figure 2), show that covalent binding to free thiol group induced irreversible structural perturbations that included increased hydrophobic exposure, a hallmark of protein denaturation and aggregation [30]. In MMTS-treated samples, denatured aggregates resistant to thermal solubilization in the presence of ionic detergent SDS could be detected by SDS PAGE. Their abundance was highest in the case of the sample treated with the highest MMTS concentration (Figure 3). Similarly to the presented results, activity loss was detected in the free thiol-involved papain capturing of gold nanoparticles [24]. Additionally, Brewer and coauthors suggested that bovine serum albumin binding to gold nanoparticles, in addition to the reaction with thiols, induces protein denaturation [40]. These results highlight the importance of optimizing MMTS treatment conditions to achieve the desired level of enzyme inactivation while minimizing the extent of protein destabilization and irreversible denaturation. A detailed analysis of bond vibrations in the Amide I region of FTIR spectra (Figure 4) and calculation of secondary structure content (Table 1) revealed exact structural changes induced by MMTS binding to the free thiol group. The detected decrease in α-helix and unordered structures content (remaining 14.6% and 28.2%, respectively) in favor of β-sheet content suggested that structural perturbations occurred in the N-terminal domain of the protein. The exact contents of remaining native-like secondary structures measured in this sample matched the calculated content of approximately 16% and 30% of α-helix and unordered structures, respectively, with the assumption that only the sequence region 47-107 (rich in short helices and unordered structures) collapsed upon MMTS binding (Figure 5). Denaturation was partially irreversible in the case of the highest MMTS concentration, indicating aggregation via newly formed aggregation-specific β-sheets. Several other studies showed the structural instability of the α-helical N-terminal domain of papain-like cysteine proteases under various conditions that were even harsher than those applied in this work. The conditions applied to the described protease destabilizations were as follows: the presence of organic solvents [39,40], chaotropes such as urea and guanidine [41], and a low [19] and high temperature [3] that led to a lowered but not completely abolished α-helix and unordered content, supporting the conclusions of this work (Figure 5). 

On the other hand, under all denaturing conditions, including those of this work, β-sheet content was unaffected or even increased, indicating the structural preservation of the C-terminal β-sheet rich domain. The C-terminal domain of papain was shown to be stable under a wide range of conditions, including changes in temperature, pH, and solvent environment [19,36,41,42], and has a certain degree of rigidity that maintains its structure and prevents unwanted conformational changes [42]. The results presented in this manuscript suggest the preservation of the C-terminal domain but also the long helix that accommodates the active site (Figure 5A) with a free cysteine sidechain that can interact with other molecules, such as ligands, substrates, or cofactors. A natural protein should have certain properties that enable it to fold into well-defined three-dimensional structures and exhibit unique chemical and physical properties. Considering the preservation of the β-sheet rich domain, papain can be a great macromolecule candidate for use as a starting point for the design of new foldamers with specific properties and functions. For example, the β-sheet domain of papain could be modified or combined with other protein domains or non-natural building blocks to create new structures with unique properties and functions, such as catalysis, sensing, or molecular recognition. 

Papain’s stable C-terminal β-sheet rich domain could potentially be used as a template molecule for molecular imprinting, depending on the desired application. This domain of papain has a well-defined three-dimensional structure and contains specific chemical functionalities, which could be used to create imprints in a polymer matrix that are complementary in shape and functionality to the β-sheet domain. These imprints could then be used to selectively recognize and bind to the β-sheet domain of papain or other proteins with similar structures or functionalities. These molecular imprinting polymers (MIPs) could be used to selectively capture and purify papain or proteins similar to papain’s β-sheet domain from complex mixtures, such as in bioprocessing or biopharmaceutical production. This could find a valuable application as the entire class of papain-like cysteine proteases counts many enzymes with great commercial value, and they can be found in different complex matrices in their source materials [43]. Also, these MIPs could be used as carriers for immobilizing enzymes with similar structures or functionalities to papain’s β-sheet domain, allowing for the enhanced stability and reusability of the enzyme in various applications. Another application could be as recognition elements in biosensors for detecting proteins, such as in disease diagnostics or environmental monitoring. Also, these MIPs could be used as carriers for the targeted delivery of drugs or other therapeutic molecules to cells or tissues that express proteins with structural and functional features resembling the previously discussed stable β-sheet domain. As papain has a wide variety of uses, these MIPs could be used for the detection of papain in food or other products or for removing papain molecules after usage.

## 5. Conclusions

Low-molecular-weight thiosulfonate derivates covalently bind to papain, acting as a reversible electrophilic thiol-blocking reagents, and in concentrations lower than 100-fold excess, they could be suitable for protease-sensing and -capturing in sensor development.

The N-terminal domain of the papain-like cysteine protease family is easily destabilized under various conditions, even in the case of mild modifications affecting only one thiol group, which could explain the activity loss, denaturation and aggregation of papain-like cysteine proteases detected in sensor material synthesis. 

The structural stability of the C-terminal β-sheet rich domain of the papain-like cysteine protease family opens up an interesting possibility to use its foldamers not only as a strategy for sensor development, but also in multiple potential applications that rely on the great commercial value of papain-like cysteine proteases.

## Figures and Tables

**Table 1:** Secondary structure content in papain samples (%).

Protein:MMTS Ratio	β-Sheet	Unordered	α-Helix	Turn	Error
MMTS 1:0	25.5	43.0	24.9	6.6	1.5
MMTS 1:1	27.9	39.5	26.6	6.0	1.4
MMTS 1:10	37.8	33.9	20.9	7.4	1.0
MMTS 1:100	52.0	28.2	14.6	7.2	1.8
DTT-MMTS 1:0	24.2	44.2	25.5	6.1	0.5
DTT-MMTS 1:1	25.2	41.9	26.0	6.9	1.0
DTT-MMTS 1:10	26.8	40.2	26.9	6.1	0.9
DTT-MMTS 1:100	30.1	38.6	24.9	6.4	1.6
X-ray	25	42	26	7	-

## Data Availability

Raw data will be available on request.

## Supplementary Materials

The following supporting information can be downloaded at https://www.mdpi.com/article/10.3390/biomimetics8030281/s1, Supplementary Figure S1: Calibration curve for Bradford assay.

## Abbreviations

ANS	8-Anilinonaphthalene-1-sulfonic acid
ATR	attenuated total reflectance
BAPNA	Nα-Benzoyl-DL-arginine p-nitroanilide hydrochloride
BSA	bovine serum albumin
DMSO	dimethyl sulfoxide
DTT	dithiothreitol
FTIR	fourier transform infrared spectroscopy
IR	infrared
MERS	Middle East respiratory syndrome
MMTS	S-Methyl methanethiosulfonate
RT	room temperature
SARS	severe acute respiratory syndrome
SDS-PAGE	sodium dodecyl sulphate–polyacrylamide gel electrophoresis
TCA	trichloroacetic acid
Tris	*tris*(hydroxymethyl)aminomethane
