# Supplementary figures and images for: Structural Transitions of Papain-like Cysteine Proteases: Implications for Sensor Development
Source: Biomimetics (Basel). 2023 Jul 1;8(3):281. doi: 10.3390/biomimetics8030281 (PMC10807080; doi:10.3390/biomimetics8030281)

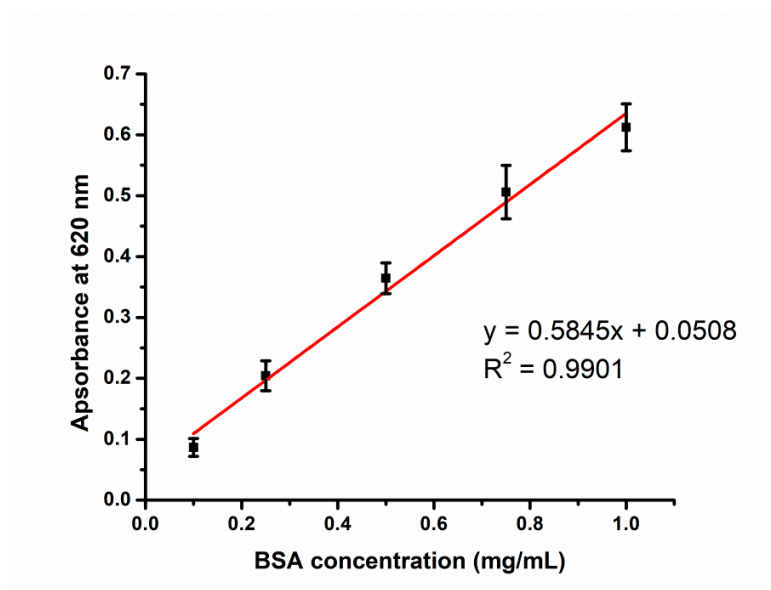

Supplementary Figure S1. Calibration curve for Bradford assay.

Supplement: Supplementary file 1 [file biomimetics-08-00281-s001.zip › biomimetics-2279760-supplementary.pdf]
